# The Impact of Emotional Feedback and Elaborated Feedback of a Pedagogical Agent on Multimedia Learning

**DOI:** 10.3389/fpsyg.2022.810194

**Published:** 2022-06-10

**Authors:** Yueru Lang, Ke Xie, Shaoying Gong, Yanqing Wang, Yang Cao

**Affiliations:** ^1^School of Psychology, Central China Normal University, Wuhan, China; ^2^Key Laboratory of Adolescent Cyberpsychology and Behavior (CCNU), Ministry of Education, Wuhan, China; ^3^No. 4 Junior Middle School of Wuhan Optics Valley, Wuhan, China

**Keywords:** emotional feedback, elaborated feedback, pedagogical agent, multimedia learning, learning processes, learning performance

## Abstract

This study aimed to explore the impact of emotional feedback and elaborated feedback provided by a pedagogical agent (PA) on learners' emotions, intrinsic motivation, agent perception, cognitive load, and transfer performance in multimedia learning. The experiment was conducted based on an actual undergraduate course. Undergraduate students (*N* = 117) were randomly assigned to one of the four conditions, where PA's feedback differed by emotional feedback (with vs. without) and elaborated feedback (elaborated feedback vs. knowledge of results). Results revealed that emotional feedback reduced learners' confusion, activated intrinsic motivation, and enhanced agent perception. In addition, elaborated feedback improved intrinsic motivation, agent perception, and transfer performance but reduced germane cognitive load. Surprisingly, there was no significant interaction between emotional feedback and elaborated feedback. These findings had implications for designing a PA with a feedback fulfilling learners' emotional and cognitive needs to maximize multimedia learning.

## Introduction

With the advance of educational technology, implementing a pedagogical agent (PA) to interact with learners in real time becomes a trend in computer-based learning environment. Providing feedback is an important function of PA, which keeps learners' motivation high and benefits performance (Dennis et al., 2015). Feedback can be classified by content into emotional feedback and cognitive feedback (Economides, 2005). Some studies have found the positive effects of emotional feedback provided by PA on emotions (Klein et al., 2002; Arroyo et al., 2011), learning motivation (Lin et al., 2014), agent perception (Woolf et al., 2010), but uncertain facilitation on cognitive processes or performance (Guo et al., 2014; Kim et al., 2017). Other studies indicated that elaborated feedback, one type of cognitive feedback, could promote academic achievement (Moreno, 2004; Lin et al., 2013; Law and Chen, 2016), while the impact on learning processes was ambiguous. The following questions need to be further solved: Can emotional feedback of PA have an impact on learning performance? What kind of cognitive feedback is more conducive to learning? Can PA incorporated with emotional feedback and elaborated feedback at the same time facilitate not only learning processes but also learning performance? On the basis of previous studies, this study aims to explore the impact of emotional feedback and elaborated feedback provided by PA on learning processes and performance and investigates whether emotional feedback and elaborated feedback can produce a mutual effect on learning.

### PA and Learning

Pedagogical agents are animated anthropomorphic characters employed in the digital learning environment to convey information and enhance motivation by simulating social interaction with learners (Kim et al., 2007; Castro-Alonso et al., 2021). There are two propositions of theories to explain how PA affects multimedia learning. One proposition was based on Social Presence Theory and Social Agency Theory. The other was based on the Interference Theory of Social Agency.

Both the Social Presence Theory and the Social Agency Theory posit that PA might be beneficial for learning processes and performance. According to Social Presence Theory, the social presence of a PA contributes to the level of intimacy that depends on factors such as smiling, dialogue, and eye contact. Therefore, learners tend to perceive the PA as a “real person” and have a more positive emotional experience, stronger learning willingness, and greater satisfaction, and then achieve better academic performance than those who do not study with PA (Gunawardena and Zittle, 1997). Another theoretical framework to explain the effect of PA on learning is Social Agency Theory. It supposes that social cues exhibited by PA make learners perceive a computer–human interaction as a human–human interaction, which primes the social interaction schema (Mayer et al., 2003; Atkinson et al., 2005). When learners interpret the learning environment as a social one, they might invest more mental efforts in processing information conveyed by PA, which in turn might improve transfer performance (Mayer and DaPra, 2012; Fiorella and Mayer, 2021).

However, the prediction of Interference Theory of Social Agency is different. It posits that PA, as a seductive detail, may hinder learners with retention and transfer performance of core materials because PA may occupy limited working-memory capacity at the selecting, organizing, or integrating stage (Paas et al., 2003; Lehman et al., 2007). Following this rationale, presenting a PA in a multimedia environment may be counterproductive.

Up to now, there is empirical evidence for Social Presence Theory and Social Agency Theory (Wang et al., 2020; Castro-Alonso and Sweller, 2021; Schneider et al., 2022). However, there is also some evidence for Interference Theory of Social Agency (Lin et al., 2020). Lin et al. (2013) and Lawson et al. (2021) pointed out that specific characteristics of agents, such as how to convey information, could affect the effectiveness of PA. Hereby, instead of generally discussing whether PA can promote learning, we should focus more on specific characteristics of PA. The necessity of a finer-grained analysis of PA's characteristics also has been suggested by Schroeder and Adesope (2014). This study investigated the effect of feedback, one of the internal PA's characteristics, on learning processes and performance.

### PA's Emotional Feedback and Learning

Feedback is one of the most important elements of instructional guidance (Panadero and Lipnevich, 2022). When a test-like event is launched, learners are encouraged to generate an answer on the basis of prior knowledge and evaluate their own current performance. Feedback is a powerful tool to help learners evaluate their learning so as to bridge the gap between current performance and the target (Hattie and Timperley, 2007).

A study by Shute (2008) revealed that feedback could not only regulate motivation and emotions, but also supply personalized scaffolding through cognitive information. In this vein, PA's feedback can be classified by content into emotional feedback and cognitive feedback. Emotional feedback aims to ameliorate learners' emotional states (Terzis et al., 2012; Tung, 2013), which can support learners through conveying inspiration, entertainment, empathy, praise, criticism, and so on (Economides, 2005). Parallel empathy and reactive empathy are the two most important forms of emotional feedback. In parallel empathy, the PA identifies and reproduces learners' emotions. In reactive empathy, the PA helps learners to regulate emotional states after identifying their emotions (Burleson and Picard, 2007; Economides, 2009; Terzis et al., 2012).

Some researchers stressed that emotions are inherently interconnected with cognition. For example, Moreno and Mayer (2007) Cognitive Affective Theory of Learning with Media makes the case that the emotional interaction between learners and computers is a motivational factor, which influences the cognitive processing of multimedia information, including selection, organization, and integration. Plass and Kaplan (2016) later proposed the Integrated Cognitive Affective Model of Learning with the Multimedia (ICALM), which proposed that emotional processes are intertwined with, and inseparable from, cognitive processes. In cognitive-emotional processing of multimedia stimuli, emotional processes make demands on cognitive resources, and cognitive activities are affected by emotional factors to some extent. Given that PA's emotional feedback is a form of human–computer emotional interaction, it may affect not only emotional processes but also cognitive processing. In this vein, a study by Schneider et al. (2022) confirmed that a PA merely with facial expressions contributed to happy and successful learning, and emotional feedback to provide learners with support was more likely to improve emotions, motivation, and performance (Kim et al., 2007).

There is mounting evidence for PA's emotional feedback that facilitates learning processes. Emotional feedback can improve learning processes by triggering positive emotions, reducing negative emotions, enhancing motivation, and bringing better agent perception. For emotions, a PA with parallel empathy or reactive empathy reduced boredom and frustration among young adults (Hone, 2006; Arroyo et al., 2011). A similar result can be found in a study with children. In Burleson (2013) study, an effective PA with parallel and reactive empathy increased positive emotions alongside a decreased sense of stuck when children solved a Tower of Hanoi problem. Regarding motivation, PA with parallel empathy enhanced university students' motivation in course of academic information seeking and course of accounting (Guo et al., 2014; Lin et al., 2014). Concerning agent perception, a study with high school students learning mathematics showed agent perception to be better when PA was featured with reactive-empathic behaviors as opposed to PA with neutral behaviors (Woolf et al., 2010). Terzidou et al. (2018) also found similar results among college students in educational virtual environment courses. For cognitive load, to our knowledge, previous studies have barely explored whether emotional feedback can affect cognitive load. Nevertheless, based on Social Presence Theory (Gunawardena and Zittle, 1997) and Social Agency Theory (Mayer and DaPra, 2012; Fiorella and Mayer, 2021), PA with emotional feedback can foster the social connection and create a human-to-human interaction atmosphere, which in turn encourages learners to invest more mental efforts in learning tasks.

In terms of learning performance, previous studies have not been able to convincingly determine whether PA with emotional feedback can promote learning achievement. Although Lin et al. (2014) and Shen (2009) have found that a PA featured with parallel and reactive empathy helped college students improve transfer performance in both accounting and mathematics, the majority of studies have revealed no significant impact on learning performance (Burleson and Picard, 2007; Kim et al., 2007, 2017; Arroyo et al., 2009; Guo et al., 2014; Terzidou et al., 2018). For instance, Kim et al. (2007) found that PA's parallel empathy did not promote pre-service teachers' retention or transfer performance in pre-service training. Guo and Goh (2016) also examined whether using an affective PA could facilitate learning. The affective PA was designed to show reactive empathy when learners get answers. The result revealed that students in affective PA condition did not perform better than those with neutral PA or No-PA in retention performance. Some researchers suggested two opposite possible reasons for these results: first, emotional feedback may not be sufficient to affect the cognition of complex learning because it does not involve supportive information for cognitive processing (Kim et al., 2007). Second, PA's emotional feedback may affect cognition, but the impact on cognitive processes is double-edged. On the one hand, emotional feedback can make the learning experience better and further expand working memory to help learners allocate cognitive resources. Learners are willing to invest more mental efforts in learning tasks (Frechette and Moreno, 2010; Plass and Kalyuga, 2019). On the other hand, emotional feedback may occupy cognitive resources, increase extraneous cognitive load, and distract attention (Li et al., 2013; Guo et al., 2014), then interfere with learning (Cabestrero et al., 2018). In order to better understand the impact of PA's emotional feedback on cognitive processing, cognitive load is necessary to be taken into consideration. Moreover, it is important to highlight that the manipulation check of emotional feedback has barely been addressed in previous studies. As a premise, the manipulation of PA's emotional feedback should be proved to be effective in this study, and then we will investigate whether emotional feedback can affect learning processes and performance.

### PA's Elaborated Feedback and Learning

Cognitive feedback serves learners with cognitive-related information targeting to support comprehension, problem-solving, and the elimination of misconceptions (Economides, 2006; Kim et al., 2007; Narciss, 2008). Further, cognitive feedback can be classified by complexity into simple feedback and elaborated feedback (Shute, 2008). Simple feedback is defined as either merely verifying whether the answer is correct called knowledge of results (KR), or showing the correct answer called knowledge of correct response (Lin et al., 2013). Elaborated feedback can provide instructional information additionally including hints, problem-solving cues, supplementary materials, etc.

As a form of cognitive feedback, elaborated feedback contains problem-solving cues and error-correcting information for knowledge construction, and so might it be able to improve outcomes of high-order learning (Moreno, 2004; Moreno and Mayer, 2005; Lin et al., 2013; Law and Chen, 2016). For instance, Moreno (2004) found that learners in the elaborated feedback group performed better in retention and transfer tests than the KR group when learning plant discovery topics. Subsequent study by Lin et al. (2013) revealed that college learners in the elaborated feedback group outperformed their counterparts in the KR group when learning physics with a PA in a computer-based environment. A meta-analysis also demonstrated that elaborated feedback was more effective in boosting high-order learning than simple feedback (Van der Kleij et al., 2014).

Furthermore, elaborated feedback is closely related to learning processes. According to the Five-Stage Model of Computer-Based Formative Assessment, learners' emotions, motivation, or cognitive aspects can be adjusted by processing and self-evaluation based on feedback (Timmers et al., 2015). Some studies have provided empirical evidence for the facilitation of elaborated feedback on motivation, agent perception, and germane cognitive load, as well as a decline in extraneous cognitive load (Moreno, 2004; Xu, 2018; Wang et al., 2019). For example, Wang et al. (2019) found that more detailed feedback resulted in lower extraneous cognitive load, more positive feedback perception, higher germane cognitive load, and stronger intrinsic motivation in psychological statistics. Nevertheless, some studies showed no significance in learning processes between students who received elaborated feedback and those who received simple feedback. One such example is reported by Lin et al. (2013), in which KR and elaborated feedback groups showed similar motivation and cognitive load during the learning of thermodynamics materials among college students.

In sum, although elaborated feedback in most previous studies could improve performance, its effect on learning processes is still uncertain. In particular, the effect of elaborated feedback on emotions seems to be a black-box. According to the Control-Value Theory of Academic Emotions (Pekrun et al., 2007), as part of a pedagogical environment, PA's elaborated feedback is posited to be antecedents of emotions, then emotions are assumed to affect motivation, cognitive resources, and achievement. With that in mind, we developed a learning system and investigated whether elaborated feedback could affect learners' emotions, motivation, agent perception, cognitive load, and learning performance in the psychological statistics course.

### The Current Study

Previous studies have revealed that PA's emotional feedback may improve learning processes, but emotional feedback alone has little effect on learning performance due to the lack of necessary cognitive support (Arroyo et al., 2011; Guo et al., 2014). Considering directly related to cognitive processing, PA's elaborated feedback is beneficial for learning performance but has uncertain impact on emotions, motivation, or cognitive load. Since emotional feedback and elaborated feedback have their own superiorities, it is necessary to further investigate whether presenting these two forms of feedback simultaneously can promote both learning processes and performance.

In the present study, we go beyond previous work by considering a manipulation check of emotional feedback to make sure the emotional feedback design was reasonable and valid. Then, we explore the impact of PA's emotional feedback and elaborated feedback on undergraduate students' learning processes and performance. Specifically, the current study explores the following question: Can PA's emotional feedback and elaborated feedback affect learners' emotions, intrinsic motivation, agent perception, cognitive load, and transfer performance? Can emotional feedback and elaborated feedback interact to influence emotions, intrinsic motivation, agent perception, cognitive load, and transfer performance?

In line with Social Presence Theory (Gunawardena and Zittle, 1997) and Social Agency Theory (Mayer and DaPra, 2012; Fiorella and Mayer, 2021), PA with emotional feedback provides a social connection that fosters social interaction schema, which in turn results in more positive emotions, stronger learning motivation, more positive agent perception, and more mental efforts invested in learning tasks (Frechette and Moreno, 2010; Plass and Kalyuga, 2019). Since cognitive activities are affected by emotional factors (Plass and Kaplan, 2016), it is hypothesized that similar results will occur in this study and the emotional feedback can facilitate learning performance.

#### Hypothesis 1

Compared to learners studying with neutral PA (PA without emotional feedback), learners who are shown PA with emotional feedback have more positive emotions, less negative emotions (H1a), stronger intrinsic motivation (H1b), better agent perception (H1c), higher germane cognitive load (H1d), and better transfer performance (H1e).

Since several previous studies have proven the learning-beneficial effects of PA's elaborated feedback (Moreno, 2004; Lin et al., 2013; Van der Kleij et al., 2014), it is assumed that a similar result will occur in this study. According to the Five-Stage Model of Computer- Based Formative Assessment (Timmers et al., 2015) and Control-Value Theory of Academic Emotions (Pekrun et al., 2007), implementing a PA with elaborated feedback leads to an increase in positive emotions, motivation, learner's perception of agent, germane cognitive load, and decrease in extraneous cognitive load (Lin et al., 2013; Xu, 2018; Wang et al., 2019). Therefore, the following hypothesis is formulated:

#### Hypothesis 2

Compared to learners receiving the knowledge of results from PA, learners receiving elaborated feedback have more positive emotions, less negative emotions (H2a), stronger intrinsic motivation (H2b), better agent perception (H2c), higher germane cognitive load (H2d), and better transfer performance (H2e).

Until now, to our knowledge no study has simultaneously investigated the intertwining influence of PA's emotional feedback and elaborated feedback. From the above literature review, PA's emotional feedback benefits learning processes, and elaborated feedback facilitates learning performance. Based on ICALM, showing these two kinds of feedback should achieve better effects on both learning processes and results. For this, the present study looks at this supposed interaction exploratory:

#### Hypothesis 3

There are interaction effects of PA's emotional feedback and elaborated feedback in terms of emotions, intrinsic motivation, agent perception, cognitive load, and transfer performance. There is an advantage of PA with emotional feedback and elaborated feedback simultaneously over other conditions in all dependent variables (H3).

## Method

This experiment investigated the impact of PA's emotional feedback (with vs. without) and elaborated feedback (elaborated feedback vs. KR) on learning, as determined by five measures: learners' emotions, intrinsic motivation, agent perception, cognitive load, and transfer performance.

### Participants

Participants were 117 undergraduate students who had finished a psychological statistics course. The average age of the participants was 19.79 (*SD* = 0.89). Of these participants, 89 were females and 28 were males. Participants were randomly assigned to one of four conditions: PA with emotional and elaborated feedback, PA with emotional feedback and KR, neutral PA with elaborated feedback, and neutral PA with KR (cf. Table 1).

**Table 1:** Distribution of participants.

	**PA with emotional feedback**		**PA without emotional feedback**		**Total**
	**PA with KR**	**PA with elaborated feedback**	**PA with KR**	**PA with elaborated feedback**	
Male	7	7	7	7	28
Female	23	22	22	22	89
Total	30	29	29	29	117

*PA, pedagogical agent; KR, knowledge of results*.

### Design of PA and Feedback

**Figure 1:**
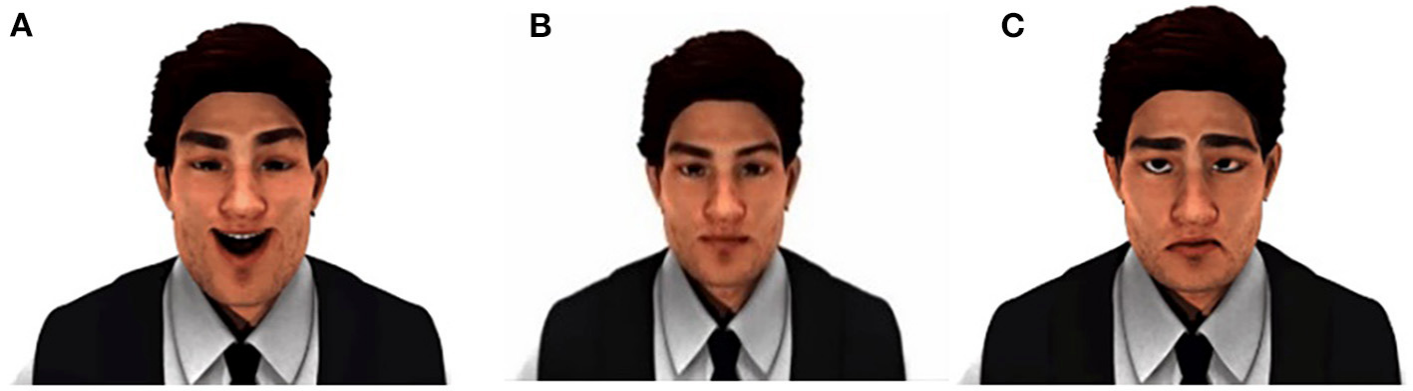
Facial expressions of PA: **(A)** pleasure; **(B)** neutral; **(C)** distress.

The PA in this study was developed using MAYA, a 3D-animation-design tool. It was designed as a male teacher with pleasant, distressed, and neutral dynamic facial expression (cf. Figure 1). The PA could nod or shake his head.

Given that confusion, boredom, frustration, enjoyment, and satisfaction were the most common emotions during online learning (D'Mello and Graesser, 2012; D'Mello, 2013), PA provided emotional feedback according to one of these five emotions every time reported by learners. In PA with emotional feedback condition, referring to a previous study (Terzis et al., 2012), PA presented parallel empathy and reactive empathy through facial expression and text feedback. The facial expression of PA was the same-valence as emotions reported by participants. That is, PA showed a pleasant expression when the participant reported a positive emotion, or a distress expression when the participant reported a negative emotion. In terms of text feedback, PA reproduced participants' emotions and provided additional supportive texts, which was referred to as Attribution Theory in previous studies (Chen et al., 2012; Cabestrero et al., 2018) to encourage participants (cf. Table 2). In PA without emotional feedback (neutral PA) condition, PA kept neutral expression all the time and did not support participants by text.

**Table 2:** Rules of emotional feedback.

	**Correct answer**	**Wrong answer**
Confusion	Text: I am sad to see you confused. This question is really difficult! Facial expression: distress	Text: I am sad to see you confused. Cheer up, never give up! Facial expression: distress
Boredom	Text: I am sad to see you boring. Let us try some challenging tasks! Facial expression: distress	Text: I am sad to see you boring. Please pay attention. Facial expression: distress
Frustration	Text: I am sad to see you frustrated. Keep going, you can make it! Facial expression: distress	Text: I am sad to see you frustrated. This question is really difficult, let's try some other tasks. Facial expression: distress
Enjoyment	Text: I am glad to see you so pleasant. I am so happy for you! Facial expression: pleasure	Text: I am glad to see you so pleasant. Keep up the good work! Facial expression: pleasure
Satisfaction	Text: I am glad to see you so satisfied. I am so happy for you! Facial expression: pleasure	Text: I am glad to see you so satisfied. Keep up the good work! Facial expression: pleasure

**Figure 2:**
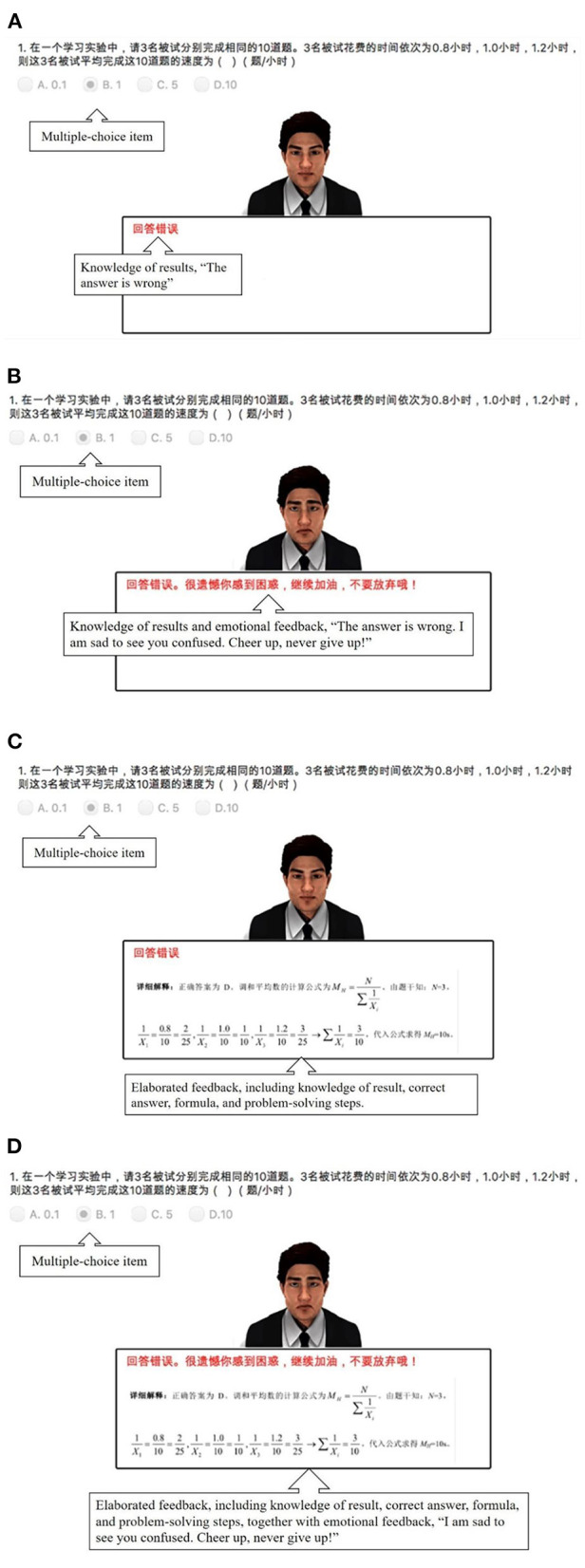
Examples for feedback presentation in the four conditions: **(A)** without emotional feedback + with knowledge of results; **(B)** with emotional feedback + with knowledge of results; **(C)** without emotional feedback + with elaborated feedback; **(D)** with emotional feedback + with elaborated feedback.

Concerning elaborated feedback manipulation, PA with elaborated feedback provided information about the correctness of the answer (e.g., “the answer is wrong”), correct answer (e.g., “The correct answer is D”), formula (e.g., “the calculation formula of the harmonic mean is $M_{N}=\frac{N}{\sum \frac{1}{X_{i}}}$”), and problem-solving steps (e.g., “according to information of the question, N=3, $\frac{1}{X_{1}}=\frac{0.8}{10}=\frac{2}{25},\;\frac{1}{X_{2}}=\frac{1}{10},\;\frac{1}{X_{3}}=\frac{1.2}{10}=\frac{3}{25}$. Thus, $\sum \frac{1}{X_{i}}=\frac{3}{10}$, substituting into formula yields:*M*_*N*_ = 10*s*”). As for PA with KR condition, PA only stated the correctness of the answer (e.g., “the answer is correct” or “the answer is wrong”). All feedback provided by PA was in Chinese (cf. Figure 2).

### Materials and Measures

#### Learning Materials and Transfer Test

The learning materials and transfer test in this study were adopted from a previous study of Wang et al. (2019), which covered psychological statistics knowledge, such as descriptive statistics, hypotheses testing, ANOVA, and probability distribution. Ten multiple-choice items were contained in the learning materials and transfer test, respectively. Each item in initial learning had a homogeneous item in the transfer test. The difficulty coefficient of items ranged from 0.4 to 0.6.

#### Manipulation Check on Emotional Feedback

Manipulation check was conducted to ensure that the manipulation of emotional feedback was effective. Participants completed a 5-point item “PA understood my emotions and provided support for me” ranging from 1 (strongly disagree) to 5 (strongly agree) (Guo and Goh, 2016).

#### Emotions

Three negative emotions (confusion, boredom, and frustration) and two positive emotions (enjoyment and satisfaction), which are mostly experienced during learning (D'Mello and Graesser, 2012; D'Mello, 2013) were measured in this study. During learning, participants reported their emotions by clicking one of the five emotion buttons after answering each item. Immediately, PA presented emotional feedback based on these self-reported emotions. Before learning, participants reported their emotions in psychological statistics classes (Münchow and Bannert, 2019). Post-test emotion questionnaire was used to measure participants' overall emotional states during learning. Participants rated 5 items (confusion, boredom, frustration, enjoyment, and satisfaction) on a 5-point scale ranging from 1 (not at all) to 5 (extremely strong) in a pre-test and post-test emotion questionnaire.

#### Intrinsic Motivation

Intrinsic motivation was measured with a 11-item scale questionnaire adapted from Instructional Materials Motivation Scale (Keller, 1983) (e.g., “I like using this system to learn”). Participants rated items on a scale of 1 (strongly disagree) to 5 (strongly agree). Cronbach's alpha for the scale in this study was 0.87.

#### Agent Perception

Participants' agent perception was measured with the Agent Persona Instrument developed by Ryu and Baylor (2003). The scale consisted of 20 items, including four dimensions: facilitating learning (10 items) (e.g., “Agent focused me on the relevant information”), credible (5 items) (e.g., “Agent was knowledgeable”), human-like (5 items) (e.g., “Agent was human-like”), and engaging (5 items) (e.g., “Agent was friendly”). All items were translated into Chinese and then back-translated to ensure equivalent meaning and double-checked by a psychological professor. Items were rated on a scale from 1 (strongly disagree) to 5 (strongly agree). Cronbach's alpha of the four dimensions and combined scale were 0.81, 0.71, 0.76, 0.85, and 0.90, respectively.

#### Cognitive Load

Perceived learning system availability, task difficulty, and mental effort were used to assess participants' extraneous cognitive load, intrinsic cognitive load, and germane cognitive load, respectively (Paas et al., 1994; Gerjets et al., 2009). Participants rated how convenient to work with the learning system ranging from 1 (extremely convenient) to 9 (extremely inconvenient), how difficult to learn psychological statistics a moment ago ranging from 1 (extremely easy) to 9 (extremely difficult), and how much effort exerted in learning ranging from 1 (extremely low) to 9 (extremely high).

### Procedures

The learning environment was programmed with Scala + Java mixed language on JVM platform, which supported the following three phases of this experiment. In the preparation phase, after completing informed consent, participants were randomly assigned to one of four conditions. Then, participants inputted demographic information and completed the emotion questionnaire. Before starting learning, participants were given two irrelevant exercises to become familiarized with the learning system. During the learning phase, after each item, participants were required to report their emotional state at that time by choosing one from five buttons representing confusion, boredom, frustration, enjoyment, and satisfaction. Immediately, PA presented emotional feedback through facial expressions and text feedback according to emotions reported by participants. The learning task lasted for 15–20 min. In the post-test phase, participants completed the emotion questionnaire, manipulation check, questionnaires on agent perception, intrinsic motivation, cognitive load, and transfer test in sequence. The experiment lasted ~30 min.

### Research Design

This study employed a 2 ×2 between-subjects design, in which independent variables included PA's emotional feedback (with vs. without) and elaborated feedback (elaborated feedback vs. KR). The dependent variables were emotions, intrinsic motivation, agent perception, cognitive load, and transfer performance.

## Results

SPSS 24.0 was used to perform the two-way multivariate analysis of covariance (MANCOVA) for emotions, prior knowledge, manipulation check, intrinsic motivation, agent perception, cognitive load, and transfer performance. Scores in the learning phase (a measure of prior knowledge) and five pretest emotions were included as covariates for manipulation check, post-test emotions, intrinsic motivation, agent perception, cognitive load, and transfer performance. The result of means and standard deviations of all variables was shown in Table 3.

**Table 3:** Means and standard deviations of all variables.

	**PA with emotional feedback (** ***M** **±SD*** **)**		**PA without emotional feedback (** ***M** **±SD*** **)**	
	**PA with KR**	**PA with elaborated feedback**	**PA with KR**	**PA with elaborated feedback**
Pretest confusion	4.30 ± 0.99	4.45 ± 1.02	4.07 ± 0.92	4.55 ± 1.02
Pretest boredom	3.37 ± 0.96	3.31 ± 0.89	3.17 ± 0.85	3.45 ± 0.87
Pretest frustration	3.43 ± 0.90	3.62 ± 0.94	3.07 ± 0.84	3.59 ± 1.09
Pretest enjoyment	3.83 ± 1.02	3.83 ± 0.66	4.28 ± 0.84	3.86 ± 0.92
Pretest satisfaction	3.93 ± 1.05	3.83 ± 0.89	4.21 ± 0.86	4.14 ± 1.13
Prior knowledge	4.40 ± 1.87	3.38 ± 1.70	4.52 ± 2.01	3.41 ± 1.66
Manipulation check	4.37 ± 1.10	4.28 ± 1.03	3.17 ± 0.76	3.79 ± 1.26
Posttest confusion	4.57 ± 1.10	4.79 ± 1.15	5.07 ± 0.53	5.14 ± 0.79
Posttest boredom	2.70 ± 0.95	2.83 ± 0.97	3.00 ± 0.96	2.90 ± 1.11
Posttest frustration	4.13 ± 1.20	4.48 ± 1.21	4.59 ± 1.02	4.66 ± 1.17
Posttest enjoyment	3.53 ± 0.97	3.14 ± 0.95	3.24 ± 0.91	3.31 ± 1.04
Posttest satisfaction	3.23 ± 1.01	3.14 ± 1.13	3.14 ± 0.92	3.03 ± 0.98
Intrinsic motivation	4.00 ± 0.60	4.29 ± 0.59	3.79 ± 0.60	4.16 ± 0.72
Agent perception	3.91 ± 0.64	4.24 ± 0.51	3.72 ± 0.54	3.96 ± 0.55
Extraneous cognitive load	5.57 ± 2.01	5.14 ± 1.96	5.24 ± 1.79	5.21 ± 2.35
Intrinsic cognitive load	7.13 ± 1.14	7.07 ± 1.28	6.93 ± 1.69	7.07 ± 1.96
Germane cognitive load	7.63 ± 1.10	7.14 ± 1.64	7.86 ± 1.06	7.28 ± 1.71
Transfer performance	5.53 ± 1.96	7.24 ± 2.13	6.14 ± 2.26	7.41 ± 2.06

*PA, pedagogical agent; KR, knowledge of results*.

### Manipulation Check

MANCOVA revealed that there was a significant difference on the manipulation of emotional feedback [*F*_(1, 107)_ = 16.76, *p* < 0.001, η_p_^2^ = 0.135]. Participants reported that PA with emotional feedback identified their emotions better and provided more support for them than neutral PA. There was no significant main effect of cognitive feedback [*F*_(1, 107)_ = 1.495, *p* = 0.22, η_p_^2^= 0.014] or interaction [*F*_(1, 107)_ = 2.89, *p* = 0.09, ${\text{η}}_{\text{p}}^{2}$ = 0.026].

### Post-test Emotions

Significant main effect was only found for emotional feedback on confusion. It could be shown that PA performing emotional feedback led to less confusion than neutral PA [*F*_(1, 107)_ = 6.66, *p* = 0.01, ${\text{η}}_{\text{p}}^{2}$ = 0.059]. However, the main effects of emotional feedback on boredom [*F*_(1, 107)_ = 1.28 *p* = 0.26, ${\text{η}}_{\text{p}}^{2}$ = 0.012], frustration [*F*_(1, 107)_ = 3.27, *p* = 0.08, ${\text{η}}_{\text{p}}^{2}$ = 0.030], enjoyment [*F*_(1, 107)_ =1.15, *p* = 0.29, ${\text{η}}_{\text{p}}^{2}$ = 0.011], and satisfaction [*F*_(1, 107)_ = 1.77, *p* = 0.19, ${\text{η}}_{\text{p}}^{2}$ = 0.016] were not significant. The main effects of elaborated feedback on emotions [confusion *F*_(1, 107)_ < 1; boredom *F*_(1, 107)_ < 1; frustration *F*_(1, 107)_ < 1; enjoyment *F*_(1, 107)_ < 1; satisfaction *F*_(1, 107)_ < 1] and interactions between emotional feedback and elaborated feedback on emotions [confusion *F*_(1, 107)_ < 1; boredom *F*_(1, 107)_ < 1; frustration *F*_(1, 107)_ = 1.03, *p* = 0.31, ${\text{η}}_{\text{p}}^{2}$ = 0.010; enjoyment *F*_(1, 107)_ = 1.77, *p* = 0.19, ${\text{η}}_{\text{p}}^{2}$ = 0.016; satisfaction *F*_(1, 107)_ < 1] were not significant.

### Intrinsic Motivation

MANCOVA found significant main effects of emotional feedback and elaborated feedback on intrinsic motivation. Compared with the neutral PA, PA with emotional feedback led to higher intrinsic motivation (marginally significant) [*F*_(1, 107)_ = 3.72, *p* = 0.06, ${\text{η}}_{\text{p}}^{2}$ = 0.034]. In a similar way, PA with elaborated feedback resulted in stronger intrinsic motivation than PA with knowledge of results (KR) [*F*_(1, 107)_ = 14.76, *p* < 0.001, ${\text{η}}_{\text{p}}^{2}$ = 0.121]. However, the interaction failed to reach significance [*F*_(1, 107)_ < 1].

### Agent Perception

Significant main effects were found on agent perception in MANCOVA for both emotional feedback and elaborated feedback. The PA with emotional feedback group perceived PA better than the neutral PA group [*F*_(1, 107)_ = 4.58, *p* = 0.04, ${\text{η}}_{\text{p}}^{2}$ = 0.041]. The same main effect occurred when PA provided an elaborated feedback while showing cognitive feedback [*F*_(1, 107)_ = 5.22, *p* = 0.02, ${\text{η}}_{\text{p}}^{2}$ = 0.046]. The interaction between these two factors was not significant [*F*_(1, 107)_ < 1].

### Cognitive Load

Only the main effect of elaborated feedback on germane cognitive load was revealed, indicating that learners reported lower germane cognitive load when they received elaborated feedback from PA [*F*_(1, 107)_ = 3.87, *p* = 0.05, ${\text{η}}_{\text{p}}^{2}$ = 0.035], but emotional feedback had no significant main effect on germane cognitive load [*F*_(1, 107)_ < 1]. In terms of extraneous cognitive load and intrinsic cognitive load, main effects of neither emotional feedback [extraneous cognitive load *F*_(1, 107)_ < 1; intrinsic cognitive load *F*_(1, 107)_ < 1] nor elaborated feedback [extraneous cognitive load *F*_(1, 107)_ < 1; intrinsic cognitive load *F*_(1, 107)_ = 2.65, *p* = 0.11, ${\text{η}}_{\text{p}}^{2}$ = 0.024] reached significance. Moreover, the interaction failed to reach significance [extraneous cognitive load *F*_(1, 107)_ < 1; intrinsic cognitive load *F*_(1, 107)_ < 1; germane cognitive load *F*_(1, 107)_ < 1].

### Transfer Performance

For transfer performance, MANCOVA only found a significant main effect of elaborated feedback. On Comparing PA with the KR group, learners in PA with an elaborated feedback condition performed better in transfer performance [*F*_(1, 107)_ = 34.79, *p* < 0.001, ${\text{η}}_{\text{p}}^{2}$ = 0.046]. Learners who received emotional feedback from PA performed similarly to those studying with neutral PA in transfer test [*F*_(1, 107)_ = 1.38, *p* = 0.24, ${\text{η}}_{\text{p}}^{2}$ = 0.013], and the interaction between emotional feedback and elaborated feedback was not significant [*F*_(1, 107)_ < 1].

## Discussion

### The Role of PA's Emotional Feedback in Learning

The present study found that PA's emotional feedback affected learning processes, such as emotions, motivation, and agent perception, whereas it had no significant effect on cognitive load or transfer performance.

Firstly, PA's emotional feedback reduced learners' confusion, which was in line with past studies (Klein et al., 2002; Prendinger et al., 2003; Hone, 2006; Shen, 2009; Woolf et al., 2010), whereas it did not increase positive emotions. H1a was partially supported. In line with Social Presence Theory, our results showed to some extent the beneficial nature of PA's emotional feedback in improving learners' emotional experience by enhancing proximity between PA and learners. In the present study, learners made 1,170 emotional reports during the learning phase, including 45% confusion, 22% enjoyment, 14% satisfaction, 14% frustration, and 5% boredom. Confusion was most reported by learners, so emotional feedback that learner received mainly targeted to reduce confusion. Other emotions appeared too infrequent to be further affected by PA's emotional feedback.

Secondly, as expected in H1b and H1c, learners' intrinsic motivation and agent perception were higher in PA with emotional feedback condition than in neutral PA condition. Consistent with Social Presence Theory and Social Agency Theory, PA's empathic behaviors created supportive atmosphere and minimized the communication barriers in human–computer interaction (Lin et al., 2013). As a result, learners perceived PA to be more intimate and were motivated to engage in learning (Guo and Goh, 2016).

However, inconsistent with H1d and H1e, PA's emotional feedback influenced neither cognitive load nor transfer performance. According to previous studies, the lack of direct useful information for cognitive processing kept learners far from constructing knowledge (Kim et al., 2007). PA's emotional feedback in this study only reproduced learners' emotions and provided emotional support so that it regulated emotional or motivational factors rather than cognitive factors. The good news was that PA's emotional feedback did not increase extraneous cognitive load or hinder learning. That is, PA's emotional feedback did not occupy learners' mental resources or act as distracting elements that drew learners' attention away from the learning content.

### The Role of PA's Elaborated Feedback in Learning

In terms of learning processes, our findings supported H2b and H2c, showing that elaborated feedback enhanced intrinsic motivation and positive agent perception. The Five-Stage Model of Computer-Based Formative Assessment suggests that learners adjust their motivational and cognitive states according to feedback received in a test-like task (Timmers et al., 2015). Elaborated feedback contained more useful information that helped learners narrow the knowledge gap between the current state and the target state. From this, learners perceived PA as a facilitator for learning, believed that they had control over their own studying, and engaged themselves in learning tasks (Pekrun and Perry, 2014; Wang et al., 2019). These results also provided supportive evidence for ICALM, where cognitive factors and motivation interweave so that elaborated feedback can support cognitive processing and motivational adjustment. Nevertheless, elaborated feedback had no impact on learners' post-test emotions, and H2a was not verified. An explanation might be that different attribution styles have opposite effects on emotions (Sixte et al., 2020). For example, when receiving elaborated feedback for correcting a wrong answer, learners with internal attribution style experience negative emotions on account of attributing failures to insufficient capability. Conversely, learners with external style attribute failure to high difficulty, so they hold the view that elaborated feedback can help them learn new things better, thereby experiencing more positive emotions and less negative emotions. For this reason, we wonder if the valences of effects of attribution styles on emotions are converse so that the total effect is not significant. Future research can bring learners' attribution style into consideration as a moderator. Besides, contrary to H2d, learners in PA with elaborated feedback group reported lower germane cognitive load compared to those in PA with KR group. It might be that since learners in PA with elaborated feedback condition were provided with detailed information for knowledge construction, they cut down on cognitive resources to process materials, i.e., they proactively reduced mental effort (Zhao, 2014) so that germane cognitive load was decreased. Given that KR only contained information about whether the answer was correct, cognitive resources were still highly required when learners needed to invest mental effort a lot in analyzing errors and correcting misunderstandings (Johnson and Priest, 2014). Although there was a significant difference between the two groups, the germane cognitive load of all learners was maintained at a high level (M > 7.28 on a 9-point scale).

As for learning performance, participants performed better in the transfer test when they received elaborated feedback than KR. The finding supported that H2e and was in line with previous studies (Lin et al., 2013; Law and Chen, 2016). In the present study, elaborated feedback not only verified whether the answer was correct, but also showed the correct answer, formula, and cues for the solution. Such cognitive information helped learners bridge the gap between old and new knowledge easier and promoted knowledge construction to the improve transfer performance (Gong et al., 2019; Wang et al., 2019).

### The Mutual Effect of PA's Emotional Feedback and Elaborated Feedback

This study did not find any significant interaction between emotional feedback and elaborated feedback on learning processes or performance. As a result, H3 was not supported. No matter which kind of cognitive feedback learners received, emotional feedback could improve learners' emotions and motivation. Containing detailed cognitive information, elaborated feedback successfully supported cognitive processing. Both emotional feedback and elaborated feedback provided means to activate learners' intrinsic motivation and had their own distinctive advantages, so their effects may be relatively stable and could not substitute for each other.

### Limitations and Future Directions

By exploring the impact of emotional feedback and elaborated feedback on multimedia learning, this study provided empirical evidence for Social Presence Theory, Social Agency Theory and ICALM. This study found that PA's emotional feedback facilitated learning processes, including better emotional experience, stronger intrinsic motivation, and enhanced agent perception; PA's elaborated feedback improved learning processes by affecting intrinsic motivation, agent perception, and finally improved transfer performance. What stands out in this study leads to strong recommendations for PA's emotional design and feedback optimization.

Three features of this work limited the conclusions we can draw about PA's emotional and elaborated feedback on multimedia learning.

First, PA's emotional feedback was not diverse enough to fully improve emotions, where emotional feedback reduced confusion but did neither reduce other negative emotions nor increase positive emotions. In this study, confusion was mostly triggered by moderately difficult tasks, and it was easiest to be affected by emotional feedback than other emotions. Nevertheless, a textual library is needed to present more personalized feedback for better experience in future studies, in which emotional feedback can be customized on learners' characteristics, current emotional states, and task performance. Besides, according to the modality principle for managing essential processing in multimedia learning (Castro-Alonso and Sweller, 2021), emotional feedback can be delivered by both auditory and visual channels (e.g., voice and facial expressions) to reduce the redundancy effect as much as possible in future studies.

Second, self-report of emotions after each item may interrupt learning. In spite of accurate identification of emotions, self-report in learning sessions scattered cognitive resources into cognitive processing of core materials and emotional awareness. Future studies can introduce artificial intelligence technology, biofeedback technology, and observation method to automate the identification of emotions. In addition, we only used self-report measures to assess learning processes. To gain more objective evidence and holistic understanding, future work should use multimodal data (the fusion of information extracted from multiple data sources, such as eye-tracking, EEG, facial data streams, physiological indexes, etc.). For example, data sources from self-report-scale and eye-tracking during learners' interaction with a learning system can be integrated and used to measure cognitive load (Sharma et al., 2022).

Third, this study was conducted with learning materials on undergraduate psychological statistics. Therefore, conclusions should be treated with prudence when generalized to other groups or courses. It will be worthwhile to repeat the experiment with learners from different grades and diverse learning materials, as well as explore whether learners' characteristics moderate the effects of feedback on learning processes and performance.

## Conclusions

This study examined the impact of PA's emotional feedback and elaborated feedback on learning. Results showed that emotional feedback decreased confusion, triggered intrinsic motivation, and enhanced agent perception, but had no impact on cognitive variables such as cognitive load and learning performance. Elaborated feedback improved intrinsic motivation, agent perception, and transfer performance but reduced germane cognitive load compared to KR. This study confirms that either emotional feedback or elaborated feedback has an essential function in multimedia learning.

## Data Availability Statement

The raw data supporting the conclusions of this article will be made available by the authors, without undue reservation.

## Ethics Statement

Ethical review and approval was not required for the study on human participants in accordance with the local legislation and institutional requirements. The patients/participants provided their written informed consent to participate in this study.

## Author Contributions

YL contributed to data collection, data analysis, and paper writing. KX contributed to experimental design and data collection. SG contributed to experimental design and paper writing. YW and YC contributed to paper writing. All authors contributed to the article and approved the submitted version.

## Funding

This study was supported by National Natural Science Foundation of China (Grant Number: 61877025) and Open Research Grant from Key Laboratory of Adolescent Cyberpsychology and Behavior (CCNU), Ministry of Education/ Hubei Key Laboratory of Human Development and Mental Health (CCNU) (Grant Number: 2019B04).

## Conflict of Interest

The authors declare that the research was conducted in the absence of any commercial or financial relationships that could be construed as a potential conflict of interest.

## Publisher's Note

All claims expressed in this article are solely those of the authors and do not necessarily represent those of their affiliated organizations, or those of the publisher, the editors and the reviewers. Any product that may be evaluated in this article, or claim that may be made by its manufacturer, is not guaranteed or endorsed by the publisher.
